# Cardio-Oncology Rehabilitation and Telehealth: Rationale for Future Integration in Supportive Care of Cancer Survivors

**DOI:** 10.3389/fcvm.2022.858334

**Published:** 2022-04-15

**Authors:** Ladislav Batalik, Katerina Filakova, Ivana Radkovcova, Filip Dosbaba, Petr Winnige, Daniela Vlazna, Katerina Batalikova, Marian Felsoci, Marios Stefanakis, David Liska, Jannis Papathanasiou, Andrea Pokorna, Andrea Janikova, Sebastian Rutkowski, Garyfallia Pepera

**Affiliations:** ^1^Department of Rehabilitation, University Hospital Brno, Brno, Czechia; ^2^Department of Public Health, Faculty of Medicine, Masaryk University, Brno, Czechia; ^3^Department of Neurology, University Hospital Brno, Brno, Czechia; ^4^Faculty of Medicine, Masaryk University, Brno, Czechia; ^5^Department of Internal Medicine and Cardiology, University Hospital Brno, Brno, Czechia; ^6^Physiotherapy Department, University of West Attica, Athens, Greece; ^7^Faculty of Arts, Department of Physical Education and Sports, Matej Bel University, Banská Bystrica, Slovakia; ^8^Department of Medical Imaging, Allergology & Physiotherapy, Faculty of Dental Medicine, Medical University of Plovdiv, Plovdiv, Bulgaria; ^9^Department of Kinesitherapy, Faculty of Public Health “Prof. Dr. Tzecomir Vodenicharov, Ph.D”, Medical University of Sofia, Sofia, Bulgaria; ^10^Department of Health Sciences, Faculty of Medicine, Masaryk University, Brno, Czechia; ^11^Department of Internal Medicine–Hematology and Oncology, University Hospital Brno, Brno, Czechia; ^12^Faculty of Physical Education and Physiotherapy, Opole University of Technology, Opole, Poland; ^13^Clinical Exercise Physiology and Rehabilitation Research Laboratory, Physiotherapy Department, School of Health Sciences, University of Thessaly, Lamia, Greece

**Keywords:** cardio-oncology rehabilitation, telehealth, supportive cancer care, cardiac rehabilitation, exercise, cancer survivors

## Abstract

The direct toxicity of cancer treatment threatens patients and survivors with an increased risk of cardiovascular disease or adverse functional changes with subsequent progression of cardiovascular complications. An accumulation of cardiovascular risk factors combined with an unhealthy lifestyle has recently become more common in cancer patients and survivors. It has been recommended to integrate a comprehensive cardiac rehabilitation model called cardio-oncology rehabilitation to mitigate cardiovascular risk. Nevertheless, cardiac rehabilitation interventions limit barriers in low utilization, further exacerbated by the restrictions associated with the COVID-19 pandemic. Therefore, it is essential to integrate alternative interventions such as telehealth, which can overcome several barriers. This literature review was designed as a framework for developing and evaluating telehealth interventions and mobile applications for comprehensive cardio-oncology rehabilitation. We identify knowledge gaps and propose strategies to facilitate the development and integration of cardio-oncology rehabilitation telehealth as an alternative approach to the standard of care for cancer patients and survivors. Despite the limited evidence, the pilot results from included studies support the feasibility and acceptability of telehealth and mobile technologies in cardio-oncology rehabilitation. This new area suggests that telehealth interventions are feasible and induce physiological and psychological benefits for cancer patients and survivors. There is an assumption that telehealth interventions and exercise may be an effective future alternative approach in supportive cancer care.

## Introduction

Cardiovascular disease (CVD) and cancer remain the world’s leading and second leading causes of death in high-income developed countries (1). The direct toxicity of cancer treatment threatens patients and survivors with an increased risk of CVD, both during acute cancer treatment and during remission and subsequent survival (2). Chemotherapy treatment is associated with adverse functional changes in the left ventricle and, depending on the dose, risk for progression of heart failure and cardiovascular complications (3, 4). Radiation therapy and its effects can cause premature ischemic heart disease. In particular, radiation therapy targeted to the thoracic part of the body may increase cardiovascular complications, as typical in lung and breast cancer (5). Anti-tumor immunotherapy has also been found to involve a higher risk of autoimmune development of pericarditis or myocarditis (6). Further long-term observation is needed to determine the effects of this new treatment on the cardiovascular system. Therapeutic monoclonal antibodies and kinase inhibitors that target the protein function of tumor cells have side effects on the cardiovascular system as well. In addition, these side effects may occur in the acute phase of treatment or the long term after treatment and cause cardiac remodeling with the progression of cardiovascular disorders (7). Therefore, there is a need to investigate the cause of cardiac remodeling and develop optimal preventive strategies.

In the population of cancer patients and survivors, cardiovascular risk factors (hypertension, diabetes, dyslipidemia) are increasing, often in combination with an unhealthy lifestyle (obesity, smoking, and reduced fitness) (8). Cancer survivors have been found to have a several-fold increased incidence of cardiovascular risk factors (9), and this condition causes a higher risk of premature morbidity and mortality. To mitigate the risk, it has recently been strongly recommended to integrate a model of comprehensive cardiac rehabilitation, also called Cardio-Oncology REhabilitation (CORE), for the population of cancer patients and survivors (10–12).

Exercise is a crucial component of supportive care to reduce cardiovascular events and is associated with a lower risk of cancer progression and improved survival after a cancer diagnosis (13, 14). Furthermore, regular exercise improves cardiorespiratory fitness (CRF), quality of life and reduces fatigue in cancer survivors (15). Among the various training modalities for cancer survivals, one can highlight exercise programs or classes either as an individual or as a group (16), including a combination of aerobic, resistance, and flexibility exercises (17), balance (18, 19), dance (20), couples-based intervention (21), likewise stationary cycle (22) or complementary and alternative medicine approach (23). Most interventions occur in hospitals, academic settings, churches, and other community-based locations (24, 25). However, cardiac rehabilitation (CR) interventions have their barriers in the form of low utilization (26), further worsened by the limitations associated with the COVID-19 pandemic and elective care limitations (27, 28). Indeed, there is a need to integrate telehealth interventions in this area, increasing access to resources and care for patients in rural areas (29).

This literature review proposes a framework for developing and evaluating telehealth interventions and mobile applications for the comprehensive CORE for patients with cancer and survivors. We discuss the role of exercise in preventing or alleviating cardiovascular events modifying CVD risk factors and CRF. Lastly, we identify knowledge gaps and propose strategies to facilitate the development and integration of telehealth CORE as an alternative to standard care approach for cancer patients and survivors.

## Key Components of the Core

**FIGURE 1:**
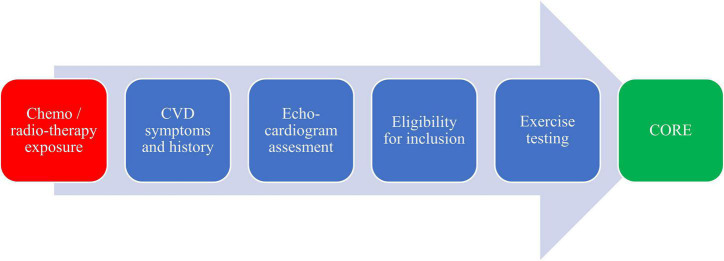
Pre-CORE process.

Recent clinical practice recommendations for preventing and monitoring the burden of treatment side effects on the heart of cancer survivors suggest that cancer patients treated with anthracyclines should be further examined and considered for risk assessment (Figure 1). The CORE model was developed to identify patients at risk for CVD, including side effects of cancer treatment on the cardiovascular system, and integrate a multicomponent CR approach to preventing and reducing cardiovascular risk (30). The individual key preventive components of the CORE are medical assessment, nutritional counseling, management of blood lipids, diabetes, blood pressure, weight management, smoking cessation, psychosocial management, exercise, and physical activity guidance (Table 1).

**TABLE 1:** Key preventive components of CORE (10, 44–46).

*Patient assessment*
• Evaluate based on medical history and cancer therapy (type, length, form of application) • Pharmacotherapy management • Identify cardiovascular risk factors and define a treatment plan (cardiac troponin, natriuretic peptides, and echocardiography) • Perform cardiopulmonary exercise test assessment
***Nutrition counseling***
• Assessment of eating habits (including objective evaluation - body mass index) • Adopt cancer-specific nutritional recommendations (referral to nutrition specialist) • Prescribe specific dietary adjustments • Incorporate behavior change models
***Blood pressure management***
• Exclusion of orthostatic hypotension by measuring blood pressure in both arms • Overview chemotherapeutic agents and molecularly targeted drugs that cause hypertension • Assess the patient’s treatment plan for blood pressure monitoring and adherence • Determinate the appropriate blood pressure target • Aggressive blood pressure targets in patients with left ventricular dysfunction
***Blood lipid management***
• Assessment of current lipid levels (high/low-density lipoprotein, triglycerides) • Determinate target values and methods to achieve them (diet, exercise, physical activity, pharmacotherapy)
***Diabetes mellitus management***
• Investigate the incidence of diabetes mellitus and review chemotherapeutic agents and molecularly targeted drugs that worsen diabetes management • Assess the presence of associated diseases and symptoms (neuropathy, chronic kidney disease, peripheral artery disease, episodes of hypoglycemia or hyperglycemia) • Examination of glycemic response to exercise (consideration of referral to diabetologist) • Determinate hemoglobin targets and methods to achieve it (diet, exercise, physical activity, pharmacotherapy)
***Obesity management***
• Assessment of the presence of obesity and overweight • Measurement of weight, body mass index, waist circumference, or targeted bioimpedance evaluation. • Determinate weight targets and a sustainable plan (lifestyle change, nutritional habits, regular physical activity) • Consultation on cancer-specific aspects that may affect body composition management
***Smoking cessation***
• Consultation of current or past use of tobacco products (including electronic cigarettes or passive exposure) • Guidance for quitting smoking (consider referral to the Nicotine Dependence Center within the institution)
***Psychosocial management***
• Identify the presence of anxiety, depression, distress, sexual dysfunction, sadness, anxiety, fear, low self-esteem, tiredness, or frustration associated with cancer and treatment • Provide support or counseling (consideration by a psychologist within the institution)
***Exercise management***
• Recommend individual prescription for an exercise involving aerobic, resistance, and flexibility forms • Assess the risk group of cardiovascular events • Assess the presence of comorbidities leading to the risk of a fall or fracture • Specify the FITT for the home-based exercise model with progressive levels, based on the cardiopulmonary exercise test • FITT prescribing should take into account individual CRF levels and muscle strength related to current cancer treatment • Determination of alternative exercise intensity using Rate of Perceived Exertion on Borg scale (0–20) at moderate to vigorous levels • Refer to a rehabilitation center for supervised exercise if available at the institution
***Physical activity guidance***
• Assessment of daily physical activity, sedentary rest period, and its quantification • Consultations on physical inactivity and its consequences • Determinate progressive and sustainable physical activity targets (up to 150 min of moderate or 75 min of vigorous-intensity activity each week)

Figure 1 illustrates a proposal of the process for cancer patients and survivors who could significantly benefit from the established multidisciplinary CORE. Patients with chemotherapy, radiotherapy, or CVD risk factors are targeted populations (31). The process represents the recommended concept for cardiovascular risk assessment (medical history and symptoms) at the CORE baseline—this combination of cardiological and oncological assessment during treatment or in remission for cancer patients and survivors. At the CORE baseline, it is essential to evaluate the safety of exercise using the cardiopulmonary exercise test to estimate the functional capacity and the presence of comorbidities associated with cancer and treatment (especially fatigue, pain, nausea, neuropathy, muscle loss, risk of falls, or depressive symptoms) (32).

The symptom-limited cardiopulmonary exercise test is the gold standard for measuring cardiorespiratory fitness in CR (33). The cardiopulmonary exercise test also facilitates assessing physiological response to exercise load, examination of cardiovascular disorders incidence, and provides limitations for prescription evidence-based exercise based on the Frequency, Intensity, Time, and Type (FITT) principle (34). Current research evidence supports the potential of exercise interventions for cancer patients to improve physical fitness (functional capacity, CRF), muscle strength, and body composition (35, 36). Indeed, patient’s exercise outcomes may differ due to side effects of the cancer treatment (fatigue, anemia) (37).

A functional capacity assessment can provide valuable information also during ongoing cancer treatment. A 6-min walk test (6MWT) is a suitable alternative to evaluate the submaximal capacity both in cardiac and cancer (38) patients before starting CR, CORE, or serially during cancer treatment (39). In several areas of cancer treatment, 6MWT to monitor patients and survivors was used for risk of decline in physical functioning during cancer treatment and subsequently in remission (40). Multidisciplinary CR centers could be an experienced potential partner in integrating CORE. However, centers may be exposed to different issues compared to the usual population of cardiac patients. Therefore, the CR team (physician, physiotherapist, or cardiovascular nurse) should be well educated and experienced in assessing the patient’s health and needs according to the specific type and possible side effects of cancer treatment. It is also appropriate to include oncological consultation in the concept of CORE programs. Table 1 displays a proposed process combining specific assessment in cancer patients and CR core/key preventive components (10).

## Recommendations for Exercise Training in Cancer Survivors

Several international organizations have issued evidence-based exercise guidelines for cancer patients and survivors. The Physical Activity Guidelines Advisory Committee (PAGAC) (41) addressed the following question: What is the relationship between physical activity and specific cancer incidence? The PAGAC evaluated 45 systematic reviews, meta-analyses, and pooled analyses comprising hundreds of epidemiologic studies with several million participants (42). The PAGAC determined that, when comparing the incidence among individuals in the highest category of physical activity with individuals in the lowest, strong evidence demonstrated reduced risks of bladder, breast, colon, endometrial, esophageal adenocarcinoma, renal and gastric cancers, with relative risk reductions ranging from approximately 10 to 20%. The World Health Organization commissioned a similar report (in 2020) (43). More than 450 studies have been conducted that have examined some aspect of physical activity and its relationship to cancer risk, and dozens of meta-analyses and systematic reviews have been published that have examined the associations for specific cancer sites.

Moreover, exercise training for survivors has been identified as a generally feasible and safe approach (44–46). Based on the guidelines, there is strong evidence for tailor-made exercise programs currently associated with the COVID-19 pandemic, supporting the need to integrate a telehealth approach (47, 48).

An example of a concept for developing an individualized exercise prescription for remotely monitored CORE cancer survivors is shown below. The FITT exercise prescription consists of a 30–60 min exercise with a frequency of three sessions per week for at least 8 weeks. The recommended aerobic exercise intensity is 60–85% of maximum heart rate based on baseline exercise testing intensity or using Rate of Perceived Exertion (RPE) in the range of 12–13 degrees of the Borg scale (0–20). The strength training prescription should include 2–3 sessions per week, with two series of 8–12 repetitions at the intensity of 60–75% of the 1-Repetition Maximum in the range of 13–15 RPE (46).

The exercise session starts with a 5–10 min low-intensity warm-up and ends with a 5–10 min cool-down low-intensity phase and stretching exercises. Usually, walking or cycling exercise modalities are recommended for the aerobic part and weights or resistance bands for the strength training. Further is essential to avoid physical inactivity and achieve 150 min of moderate to vigorous-intensity aerobic activity (46). Indeed, the FITT exercise prescription should respect current CRF levels, cancer treatment, cardiovascular risk of exercise-related events, and the risk of falls or fractures of the patients. The initial dose of exercise and additional sessions should be progressive. Finally, behavioral change strategies target achieving a sustainable and long-term exercise regime (49).

## Integration of Cardiac Rehabilitation in Supportive Cancer Care

One of the leading causes of death among cancer survivors is CVD (50). Long-term follow-up studies of cancer survivors in adults have stressed CVD as a competing risk and the necessity for pre-treatment CVD risk factor assessment, monitoring during treatment, and post-treatment care (51–53). Practical and feasible therapies are required to reduce CVD risk in cancer patients. A delivery approach similar to CR programs could be feasible (10, 54). Cancer rehabilitation programs are proactive approaches to the physical and/or functional decline of the patients associated with cancer or the cancer treatment process (55). On the other hand, CR is described as “the provision of comprehensive long-term therapies comprising medical evaluation, prescription exercise, cardiac risk factor management, and education, counseling, and behavioral interventions,” according to the American Heart Association (AHA) and has become an evidence-based practice guideline for the secondary prevention of ischemic heart disease (10, 11, 56, 57). Although CR is a cost-effective therapy (58, 59), it is underutilized (60–63), programs in place to improve referral and participation by including virtual models for CR outside of the traditional center-based approach (64). As a result, a scientific rationale for integrating cancer rehabilitation and CR is urgent to achieve the growing clinical needs for cancer-related or cancer-treatment-related CVD (10). The existing infrastructure of CR was acknowledged as a suitable model for cancer patients and survivors. The AHA introduces the integration idea and recommends the CORE framework (10). In addition, exercise may be beneficial in some types of cancer (breast, colon, lung, prostate, lymphomas, and other hematological diseases) when the associated cardiovascular risk is not negligible enough to exploit CR’s therapeutic action fully. Finally, improving CRF may provide benefits in terms of cancer-related symptom alleviation, mortality reduction, and/or cancer recurrence (10, 65, 66). Cancer treatment induces (besides direct cardiotoxicity) potential damage to the cardiovascular system by causing hormone deficits, alterations in insulin sensitivity, lipid metabolism, and inflammatory state. By fostering the development of metabolic syndrome, these alterations can exacerbate cardiometabolic risk (67, 68). The CORE is the ideal strategy for the prevention of these disorders. In subjects with a history of CVD, fasting total cholesterol, high/low-density lipoprotein, and triglyceride levels should be assessed during CORE, with a target of a low-density lipoprotein level below 70 mg/dL (66). Although a CVD risk score is beneficial (69), cancer-related variables such as chest and mediastinal irradiation must be considered when customizing individual cardiovascular risk (52, 70). The CORE team will create a customized aerobic and resistance training program to meet the target objectives.

## Telehealth Core Feasibility for Cancer Patients and Survivors

Recently, a growing trend has been identified in studies addressing the feasibility, safety, and effect of telehealth exercise interventions in delivering supportive care for cancer patients and survivors (Table 2); only nine studies were included for this review, which illustrates the work’s “early” nature.

**TABLE 2:** Characteristics and results of studies that evaluated the effects of home-based exercise intervention.

Study	Treatment	Cancer	N	Weeks	Exercise program	Intensity	Frequency	Monitoring/feedback	Results	Adverse events	Adherence
Alibhai et al. (73)	During ADT	PC	59	24	Aerobic, resistance and flexibility; 150 min/wk	60–70% HRR, 3–6 RPE(10)	4–5/week	HR monitor/phone call	Feasibility ↑cardiorespiratory fitness ↑PRO	2 cases mild grade no events grade 3 or higher	50%
Ariza-Garcia et al. (74)	During CT	BC	68	8	Aerobic and resistance; 15–30 min/session	45–60% HRmax	3/week	Web-based intern platform/video call	↑6MWT distance ↑muscle strength	NR	73%
Cornette et al. (75)	During ADT	BC	44	27	Aerobic and resistance, 30–50 min/session +	1VT	3/week	HR monitor; exercise diary/phone call	Feasibility ↑cardiorespiratory fitness ↑PRO	NO adverse events	88%
Galiano-Castillo et al. (72)	Post CT, RT, surgery	BC	81	8	Aerobic and resistance	NS	3/week	Web-based intern platform/video call	↑6MWT distance ↑muscle strength ↑PRO	NO adverse events	94%
Gehring et al. (76)	Post CT, RT or surgery	GLIOM	34	24	Aerobic exercise	60–85% HRmax	3/week	HR monitor; training log online/e-mail	Feasibility ↑cardiorespiratory fitness ↑PRO	NO adverse events	79%
Hvid et al. (77)	Post surgery	PC	25	96	Aerobic exercise; 45 min/session	60–65% VO_2_max	3/week	HR monitor; training log/in person visit	Feasibility ↑cardiorespiratory fitness ↑body composition	NR	88%
Cheville et al. (78)	During or post	different types	516	12	Walking and resistance	NS	5/week	Telephone call	↑PRO	NO adverse events	NR
Lahart et al. (79)	Post ADT or surgery	BC	80	24	Aerobic exercise; 30 min/session	NS	Gradually 3–7/week	PA diary/phone call	↑cardiorespiratory fitness ↑PA levels	NR	NR
McNeil et al. (80)	Post CT, RT, surgery	BC	45	12	Aerobic exercise; LIG: 300 min/wk, HIG: 150 min/week	LIG: 40–59% HRR; HIG: 60–80% HRR	NS	HR monitor; exercise diary/phone call; email	Feasibility ↑cardiorespiratory fitness ↓sedentary time	NR	100%

*CT, chemotherapy; RT, radiotherapy; HRR, heart rate reserve; HR, heart rate; VO_2_max, maximal oxygen consumption; ADT, androgen deprivation therapy; RPE, rating of Perceived Exertion; VO_2_peak, peak oxygen consumption; HRmax, maximum heart rate; PA, physical activity; VT, ventilatory threshold; LIG, low intensity group; HIG, high intensity group; 6MWT, 6 min walking test; NR, not reported; PRO, patient reported outcomes; BC, breast cancer; PC, prostate cancer; GLIOM, gliomas.*

For example, Galian-Castillo et al. (71) reported that among the 81 breast cancer survivors, aerobic and resistance exercises performed three times a week under telerehabilitation supervision led to a significant improvement in 6MWT (*p* < 0.001), quality of life (*p* < 0.001), pain severity (*p* < 0.001), and fatigue (*p* < 0.001) (71, 72). In another study, prostate cancer survivors who participated in home-based aerobic exercise three times a week with regular teleconsultations significantly improved CRF (*p* < 0.01) and body composition in the form of fat loss (*p* < 0.05) (73). Furthermore, Ariza-Garcia et al. (74) reported that during chemotherapy treatment, in 68 patients with breast cancer who participated in an 8-week Web-Based Exercise System intervention improved 6MWT (*p* < 0.05) and abdominal, back, and lower body strength (*p* < 0.001) (74).

**FIGURE 2:**
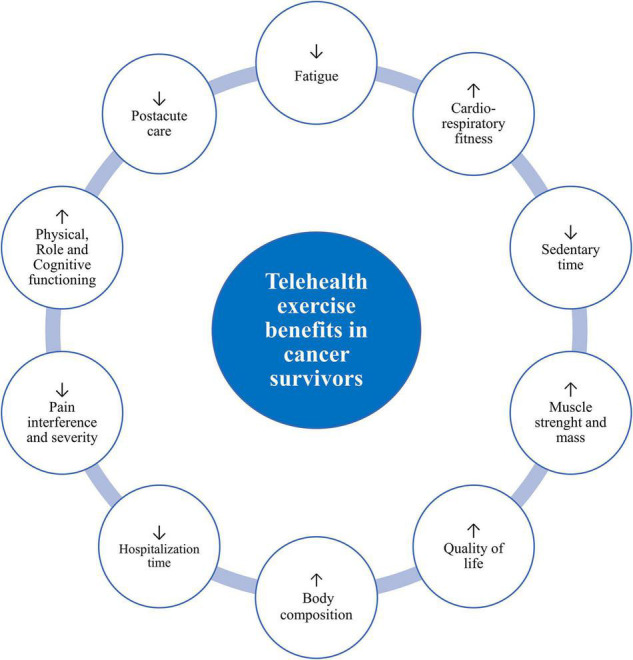
Potential telehealth exercise benefits in cancer patients and survivors. Exercise training can be an effective intervention for attenuating treatment and treatment-related cardiovascular disease prevention.

In summary, telehealth exercise and/or physical activity intervention models are feasible, safe, and have shown good adherence. The findings from this review suggest that exercise can provide a variety of benefits for cancer survivors during the rehabilitation period after discharge from the hospital (Figure 2). Due to the combined effects of cancer treatment, which increase the risk of morbidity and mortality, there is good reason to identify at-risk groups of patients and survivors who provide individualized exercise interventions, especially those with barriers to accessing centralized services.

## The Target Population for Core

There are no consensus recommendations for therapies to prevent all cancer treatment-related CVD risks. Patients with multiple cardiovascular risk factors, including patients at high risk for cancer treatment (radiotherapy or high-dose chemotherapy) and adult survivors after high-exposure childhood cancer treatment, are likely to benefit the most from CORE (81). Recent AHA scientific statements aimed at preventing and managing cardiac dysfunction of post-treatment cancer patients and survivors identified evidence-based recommendations for selected cancer therapies and exposures predisposing to cardiac dysfunction (10). Indeed, referenced guidelines are only a starting point for recommendations. Multidisciplinary expertise cooperation (internal, cardiology/oncology) is required to assess the risk of cancer treatment-related CVD, which will vary depending on the type and intensity of treatment, medical history, cardiovascular risk factors, and patient age.

Patients who meet one of the criteria listed below are considered high risk for cancer-related CVD:

• Treatment with high doses of anthracyclines (doxorubicin ≥ 250 mg/m^2^)
• High-dose radiation (≥30 Gy if targeted to the chest)
• Combination: low doses of radiation and low doses of anthracyclines
• Combination: treatment with low doses of anthracyclines plus ≥ 2 cardiovascular risk factors (hypertension, diabetes, dyslipidemia, metabolic syndrome, obesity, smoking) plus older age (60+) or presence of CVD (history of coronary heart disease, cardiac failure, reduced left ventricular function)
• Treatment with lower doses of anthracyclines followed by trastuzumab

The rationale for comprehensive telehealth CORE Until now, the focus of cancer survivors has been investigating the presence of cardiotoxic-induced cardiac dysfunction. However, the side effects of cancer treatment extend beyond the cardiovascular system. Indeed, it has been found that CRF and assessment of cardiopulmonary function may have prognostic significance in cancer patients (82, 83). Metanalysis has shown that CRF decreases during exposure to combination anticancer treatment (84), and development may not reverse after treatment. Low CRF has been identified in several survivors, such as lung, cervical, endometrial, and ovarian cancers or young adult cancer survivors (85–88). Thus, a growing need to include an exercise training strategy to prevent CRF decrease during anticancer treatment (89). Exercise training is one of the most important components of comprehensive CR, leading to increases in CRF and reducing cardiovascular morbidity and mortality (90, 91). Therefore, there is an assumption that the implication of implementing comprehensive CORE may represent significant physiological and psychological benefits for cancer patients and survivors.

Recently, several studies have integrated CR exercise interventions and demonstrated effectivity on CRF and other cardiovascular outcomes in various cancer populations during and after primary adjuvant therapy. A meta-analysis of 1990 patients intervened by exercise training after completion of cancer treatment recorded a significant increase in CRF compared to 1,642 patients in usual care by peak oxygen consumption + 2.13 ml/kg/min (95% CI, 1.58–2.67 ml/kg/min; *p* < 0.001) (92). In short, exercise training can positively affect the decrease in CRF induced by cancer treatment and improve CRF upon completion of cancer treatment. Developing and implementing novel approaches, such as telehealth CORE, would increase participation utilization by expanding capacity (93, 94). Another essential benefit is cost-effectiveness, especially for patients requiring direct exercise supervision (95). The telehealth CORE provides components that are comparable to the supervised center-based CORE. However, there are limitations in that CR staff are not comprehensively trained in cancer treatment and supportive care. Indeed, the responsibility for identifying and referring cancer patients at risk for cardiac dysfunction remains in the hands of oncologists and internal medicine physicians. Although cardio-oncology collaboration usually begins with cardiovascular disorders, a proactive clinical specialist collaboration is needed to develop a comprehensive CORE.Lastly, the World Health Organization statement also recommended following the patient-centered approach and considering individualized treatment and patient preferences in the telehealth interventions (96). The theory assumes that prescription adherence can be improved by designing intervention programs according to the preferences and needs of patients. Indeed, it was identified that patient preferences could affect treatment outcomes (97). In addition, a recent study found that eligible patients for CR were given the choice of a home-based or center-based intervention, with almost half preferring a home-based approach (98). Therefore, evaluating patient preferences is essential to developing comprehensive patient-centered strategies.

Key activities and strategies for telehealth CORE include: (i) identifying patient preferences (can be individual at different stages of treatment), (ii) determining safety by assessing the risk of exercise-related adverse events, (iii) secure communication links with the patient versus CORE center (smartphone apps, web-based-platforms, wearable tracking systems, and sensors), (iv) home-based exercise prescription (with a particularemphasis on individual response to dose and type of exercise), and (v) provide patient self-directed plan feedback (monitoring exercise progress, adherence to exercise prescription and nutrition plans, motivational support and adaptations).

## Research Gaps and Future Directions

According to the latest findings, telehealth and mobile technology-based interventions should incorporate individual patient assessment, exercise training, physical activity consultation, self-management of modifiable risk factors, and psychological counseling (99, 100). Nevertheless, the optimal combination and importance of the individual preventive components required to maximize the effect of telehealth and mobile applications remain unclear and form a crucial field for future research (101).

Secondly, regular physical activity improves cardiovascular health outcomes. Although a questionnaire survey is a standard method of assessing physical activity, it was found to show a high degree of variability compared to objectively measured physical activity (102). Thus, mobile technologies and telehealth provide a suitable alternative, as physical activity can be recorded and reported on real-time platforms. In addition, mobile devices provide opportunities to interface with pedometers, wearable sensors, accelerometers, and other wireless technologies that track physical activity and exercise.

It is also essential to consider the psychological needs of cancer patients. The prevalence of distress in patients with cancer ranges from 35 to 55%. Lack of control over treatment decisions also plays a part in the onset of depression, sadness, anxiety, and decreased quality of life. Preliminary reports indicate that home-based exercise interventions may provide physiological and psychological benefits for cancer survivors (103). However, limited studies have been conducted on modern technology in the psychotherapeutic process. It has also been shown that stressed patients tolerate therapy less well, while motivated patients who adhere to the rehabilitation program achieve better health results. The complementation of rehabilitation with immersive virtual reality therapy systems or psychotherapeutic teleconsultations may provide vital support for patients. However, specific requirements must be met for remote systems to help improve the mental state of patients. Such a system should address therapeutic factors such as breathing exercises, relaxation, mindfulness, or elements of psychotherapy.

Telemental health is currently the most widely used telemedicine option. Indeed, visualization as a therapeutic method may boost the immune system (104). This technology has gained the most popularity and effectiveness in treating depression, anxiety disorders, and post-traumatic stress disorder (105). Thus, virtual technology supplemented with telemental health projects has the potential to make healthcare more efficient.

Moreover, further investigations could be integrated into a comprehensive, remotely guided examination with other wearable sensors such as heart rate, ECG, blood pressure, body weight, or glycemia. Future perspectives and research should aim to analyze the validity and reliability of sensors and develop valuable benchmarks for assessing outcomes in the prevention and supportive cancer care associated with mobile technologies.

Furthermore, there is a need to address concerns related to the legal clarity of telehealth services provision. It is also unclear what effect telehealth and mobile technologies will have on the overall cost of supportive cancer care (106). While mobile technology and information and communication technology services can be costly in terms of direct cost, in the beginning, the potential benefits of efficacy and acceptability on the patient side can provide more significant benefits.

Finally, analyses and large-scale feasibility evaluations are needed to prevent patients from dropping out of telehealth interventions. Therefore, conducting clinical feasibility evaluations of the telehealth or mobile app concept is recommended using usability testing and field studies to analyze qualitative and quantitative characteristics of efficacy, safety, adherence, and user satisfaction.

## Conclusion

Cancer patients and survivors are under-represented in current cardiovascular prevention studies. Given the available evidence on CVD risk in cancer patients and the possible risk reduction through CORE, there is a need to develop patient-centered interventions to increase participation and utilization. Although there is limited evidence, pilot results support the feasibility and acceptability of telehealth and mobile technologies in CORE cancer patients and survivors. This new area suggests that telehealth interventions can induce physiological and psychological benefits in cancer patients and survivors. Therefore, telehealth intervention and exercise may be an alternative approach to standard cancer care in the future.

## Author Contributions

All authors listed have made a substantial, direct, and intellectual contribution to the work and approved it for publication.

## Conflict of Interest

The authors declare that the research was conducted in the absence of any commercial or financial relationships that could be construed as a potential conflict of interest.

## Publisher’s Note

All claims expressed in this article are solely those of the authors and do not necessarily represent those of their affiliated organizations, or those of the publisher, the editors and the reviewers. Any product that may be evaluated in this article, or claim that may be made by its manufacturer, is not guaranteed or endorsed by the publisher.
